# Anti-filarial immunity blocks parasite development and plays a protective role

**DOI:** 10.1371/journal.pone.0199090

**Published:** 2018-06-21

**Authors:** Prakash Kumar Sahoo, Santosh K. Panda, Ashok Kumar Satapathy, Sanghamitra Pati

**Affiliations:** 1 Division of Immunology, ICMR-Regional Medical Research Centre, Chandrasekharpur, Bhubaneswar, Odisha, India; 2 Medimmune, One Medimmune Way, Gaithersburg, MD, United States of America; Centro de Pesquisas Rene Rachou, BRAZIL

## Abstract

Lymphatic filariasis is a complex parasitic disease having a spectrum of clinical parameters which are critical in deciding the severity of the pathogenesis. Individuals residing in the endemic areas are categorized into different clinical groups such as: EC (endemic controls-free of disease and infection), AS (asymptomatic carriers- free of disease but carries both antigens and microfilaria (Mf) in circulation), CR (cryptic-free of disease and Mf but having circulatory antigen) and CH (chronic-having manifestations of elephantiasis and hydrocele). The immune response to the parasitic infection is well studied, whereas the protective mechanism explaining the fate of antigenemia and filaremia between AS and CR group remains unexplained. Increased anti-Mf antibodies have been implicated for Mf clearance in experimental infection models whereas its role in clinical filariasis is not known. Here, we followed up two groups of 24 and 33 CR cases for 18 and 36 months respectively and analyzed both the clinical parameters and the anti-filarial antibody response. The humoral response to both whole filarial antigen and Mf antigens as well as recombinant active parasitic antigens was significantly higher in CR cases than AS individuals, whereas the clinical parameters remain unchanged. This study made insights into the protective immune mechanism responsible for the clearance of Mf from circulation in CR individuals.

## Introduction

Lymphatic filariasis (LF) caused by *Wuchereria bancrofti* is a major public health problem contributing to about 90% of the 120 million people affected across the globe [1,2,3]. In India, 23 states /UTs are known to be endemic to LF and 553 million people are at risk of infection with 31 million parasite carriers and 23 million with symptomatic filariasis [4]. Clinical manifestations such as lymphedema, elephantiasis and/or hydrocele are associated with this disease. Based on disease manifestations, presence of Mf and filarial antigen in circulation, people residing in endemic areas have been classified into different clinical categories, such as: EC (endemic control -free of disease and infection), AS (asymptomatic carriers- free of disease but having antigens and microfilaremia in circulation) and CR (free of disease, free of Mf but having circulatory filarial antigen, CFA) and CH (disease with CFA) [5]. Although immune-modulation indicated by the filarial parasite is well studied, the protective mechanism responsible to decide the fate of microfilaremia between AS and CR group remains unsolved in clinical filariasis.

Humoral immunity operated against Mf sheath have long been implicated for protective mechanism against filariasis. A reverse association between the level of antibodies against Mf sheath and microfilaremia has been demonstrated in experimental models and clinical filariasis [6,7]. Immune response against dead Mf was found to be protective against later implanted Mf [8,9]. Furthermore, anti-Mf IgG, even serum of *B*. *pahangi* immunized animals were found to be protective against Mf [10]. All of these studies suggested that immune responses to Mf play a major role in clearance of the parasite from the host. Since no suitable model is available to mimic clinical filariasis, we studied the immune response against the Mf in naturally infected clinical individuals. 57 cryptic individuals were followed up, to study the anti-Mf immunity for 18 and 36 months.

In this study, clinical parameters, disease status, microfilaremia, and antigenemia were monitored. The antigenemia remained unchanged during this course of time whereas the antibody levels against the crude filarial antigen, Mf antigen, and recombinant antigen were significantly higher in CR group as compared to AS group.

## Materials and methods

### Ethics statement

Approval for the study was obtained from the institutional ethical committee of theRegional Medical Research Center (Indian Council of Medical Research), Bhubaneswar,Odisha. Adult human blood was collected from healthy donors following written informed consent.

### Collection of human blood samples

The study was carried out in two villages, namely Kanapur and Jatni area of Khurda district of Odisha, India, known to be endemic for lymphatic filariasis [11]. A mass blood survey was carried out from June 2002 to December 2003 to know the filarialsis status. DEC consumption status by acute and/or chronic individuals was recorded.

Approximately 5 ml of blood was collected from each person, those who volunteered to give blood samples. One ml blood was used for Mf identification. Serum samples were separated from cells in the laboratory and frozen at -20°C. Approval for the study was obtained from the institutional ethical committee of the Regional Medical Research Center (Indian Council of Medical Research), Bhubaneswar, Odisha, and informed consent was given by the study subjects for the collection of blood samples.

### Follow up of CR patients

24 cryptic individuals from Kanapur (Cohort A) and 33 cryptic individuals from Jatni (Cohort B) were followed up for 18 months and 36 months respectively. 30 AS patients from both the villages were recruited for comparison. They were asked for their symptoms and blood samples were collected for parasitological and immunological investigations.

### Preparation of soluble filarial antigens

PBS-solubilized extracts from adult and Mf stage of *S*. *digitata* were prepared as described by us elsewhere [12,13], and the recombinant antigens were donated by Prof. R.M. Maizels from University of Edinburgh, London. All of the solubilized antigens were frozen at -20°C for further use.

### Circulating filarial antigen assay (Og4C3)

Circulating filarial antigen (CFA) levels in serum samples were measured as described by us elsewhere [13], using a TropBio ELISA Kit (Tropical Biotechnology) according to the manufacturer’s protocol. Levels are expressed as arbitrary antigen units determined according to internal standards provided in the kit.

### ELISA

Polystyrene plates (96 well, MaxiSorp; Nunc) were coated with 1–2µg/ml of *S*.*digitata* adult antigen, Mf antigen or recombinant filarial antigens (i.e. ALT, VAL. SPN and CPI) and incubated for 5hrs at 37°C and transferred to 4°C for overnight incubation. After the plates were washed with PBS, wells were blocked with 1% skimmed- milk- PBS for 2hrs. Then 200-fold diluted human filarial serum was incubated and plate bound IgG was detected by using 1000- fold-diluted peroxides-labeled anti–human IgG. The enzyme activity was measured using orthophenylene diamine (P1526; Sigma). Absorbance was recorded at 492 nm, and the results are expressed as arbitrary ELISA units.

### Immunofluorescence assay

Immunofluorescence assay to detect IgG against the surface of Mf was performed as per procedure previously described [14], with the following modifications. Human serum, diluted 10-fold in PBS containing 0.1% bovine serum albumin, was applied to microscopic slides with fixed Mf and then incubated for 2.5 hrs in humid chambers. The slides were washed in PBS and treated with 100-fold- diluted FITC-conjugated anti–human IgG in the same buffer. The reactivity was recorded using fluorescence microscope.

### Data analysis

Student’s t test or the X2 test was applied to determine the statistical significance between different groups. All statistical tests were performed using Graph Pad Prism software (version 4.0). Differences were considered significant at P< 0.05.

## Results

### Study population

**Fig 1:**
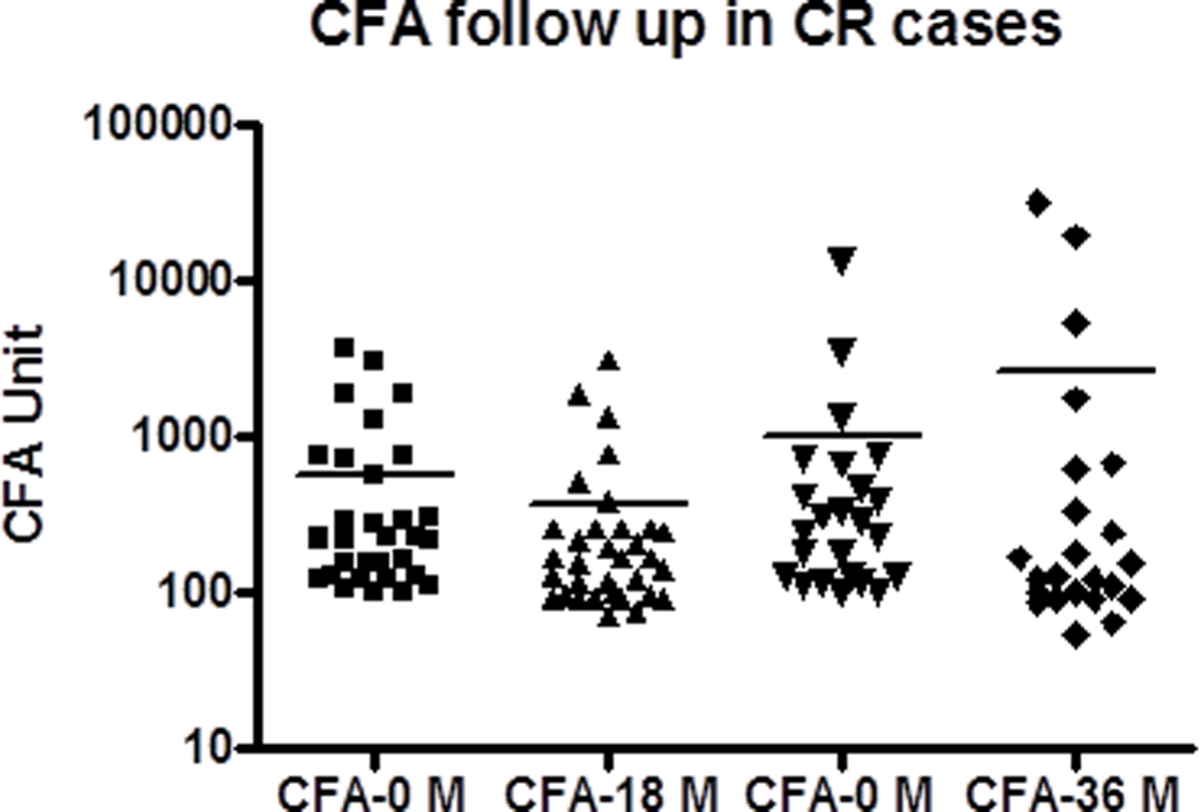
Comparison of CFA between AS and CR cases. CFA levels of 30 asymptomatic Mf carriers and 31 cryptic individuals were taken in to consideration for comparison. They are age sex matched and other parameters has been taken care as narrated in the Table 2.

The cryptic individuals were followed up in both the villages. Cohort A (24 individuals) and cohort B (33 individuals) were rechecked for filariasis after 18 and 36 months respectively. Clinical history was recorded and, blood samples were collected for Mf and CFA. There was no significant change in clinical manifestations, Mf status (Table 1) and CFA density (Fig 1) after the time period. Only one patient from each cohort converted to MF carrier. The geometric mean intensity (GMI) of CFA at two different time points was not significantly different.

**Table 1:** Mf status of cryptic individuals followed up for 18 and 36 months in village Kanapur and Jatni.

	Time ‘0’ (%)	18 Months (%)	36 Months (%)	Total (%)
**Cohort A**	0/24 (0)	1/24 (4.16)	ND	1/24 (4.16)
**Cohort B**	0/33 (0)	ND	1/33 (3.0)	1/33 (3.0)
**Total**	**0/57 (0)**	**1/24 (4.16)**	**1/33 (3.0)**	**2/57 (3.508)**

### Microfilaremia and antigenemia remain unchanged in cryptic individuals

**Fig 2:**
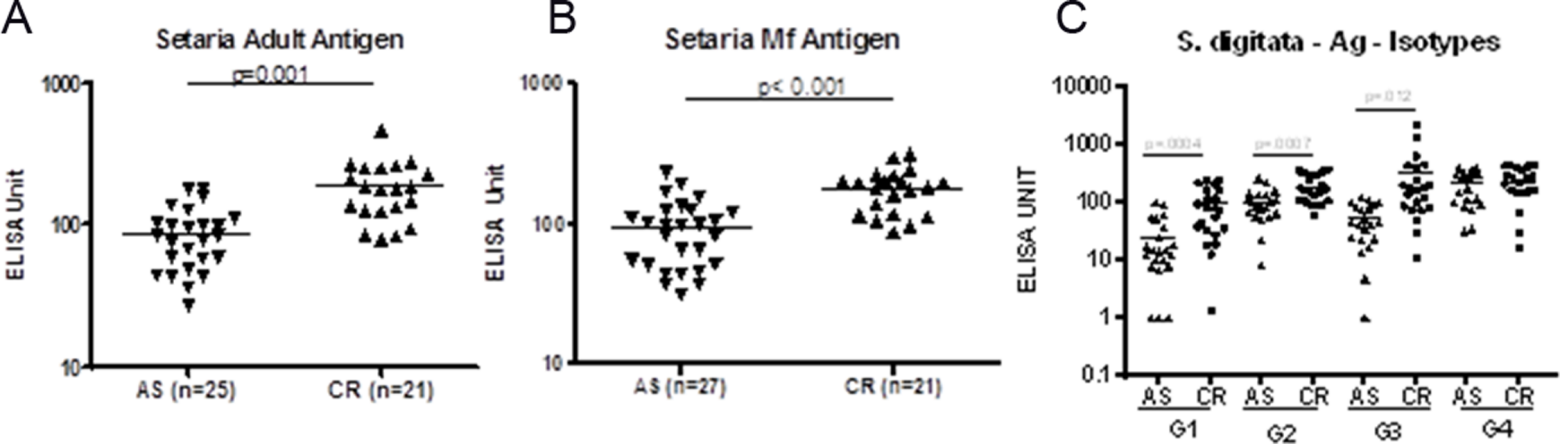
Profile of IgG and IgG subclasses against different filarial antigens. Plasma levels of IgG and IgG subclasses were analyzed by ELISA. IgG against whole somatic antigen of adult Setaria worm (A) and Setaria MF (B) were analyzed. IgG isotypes against somatic antigen of adult setaria worm were analysed (C). Mean of 30 and 31 samples were shown.

Clinical parameters, microfilaremia, and antigenemia levels were analyzed in both the cohorts. It was observed that the above parameters remain unchanged during the course of time. Neither they acquired active infection nor the levels of antigen in circulation changed significantly (Tables 1 and 2). These observations suggested that, the immune mechanism responsible for the elimination of active microfilaremia remains effective during the time. This finding led us to investigate the levels of IgG antibodies against the Mf sheath. Anti-sheath IgG antibody levels in CR cases were significantly higher as compared to AS cases (Fig 1). However, only 16% of the cases exhibit antibody positivity (Table 3). This result suggested a role of additional protective mechanism in play. Then we analyzed the IgG levels against the crude adult antigen and Mf antigen in both the groups. IgG levels against both the antigens were significantly higher in CR group as compared to AS group (Fig 2A and 2B). The above observations suggested that the antibodies against the filarial antigen and Mf antigen play a potential role in Mf clearance.

**Table 2:** Details and intensity of infection in two study group.

Sl.No.		AS	CR
1.	Sample No. (n = 61)	30	31
2.	Male/Female	21/9	23/8
3.	Median age in years (Range)	26(9–66)	28(7–75)
4.	Mf Status	30/30	0/31
5.	Mf density (GMI/ml)	2194	NA
6.	CFA Status	29/30	31/31
7.	CFA Density–GMI	27056	949
8.	Clinical Symptoms	No	No

**Table 3:** Anti-sheath antibody between AS and CR cases.

	AS	CR
**ASAB+VE**	2	10
**ASAB-VE**	57	52

P = 0.0191; χ^2^ = 5.492

### Profile of IgG subclass in AS vs CR group

The role of different IgG antibody subclasses against filarial antigens in filarial pathogenesis has been demonstrated. Increased levels of IgG3 antibodies in individuals with active infection were observed as compared to endemic controls [15,16]. Therefore, we investigated the profile of different IgG subclasses against filarial antigens in AS and CR cases. An increased level of IgG1, IgG2, and IgG3 antibody levels were observed in CR cases as compared to AS, whereas no difference in IgG4 was observed (Fig 2C).

### The IgG antibody levels against recombinant filarial antigens is significantly more in CR cases as compared to AS

**Fig 3:**
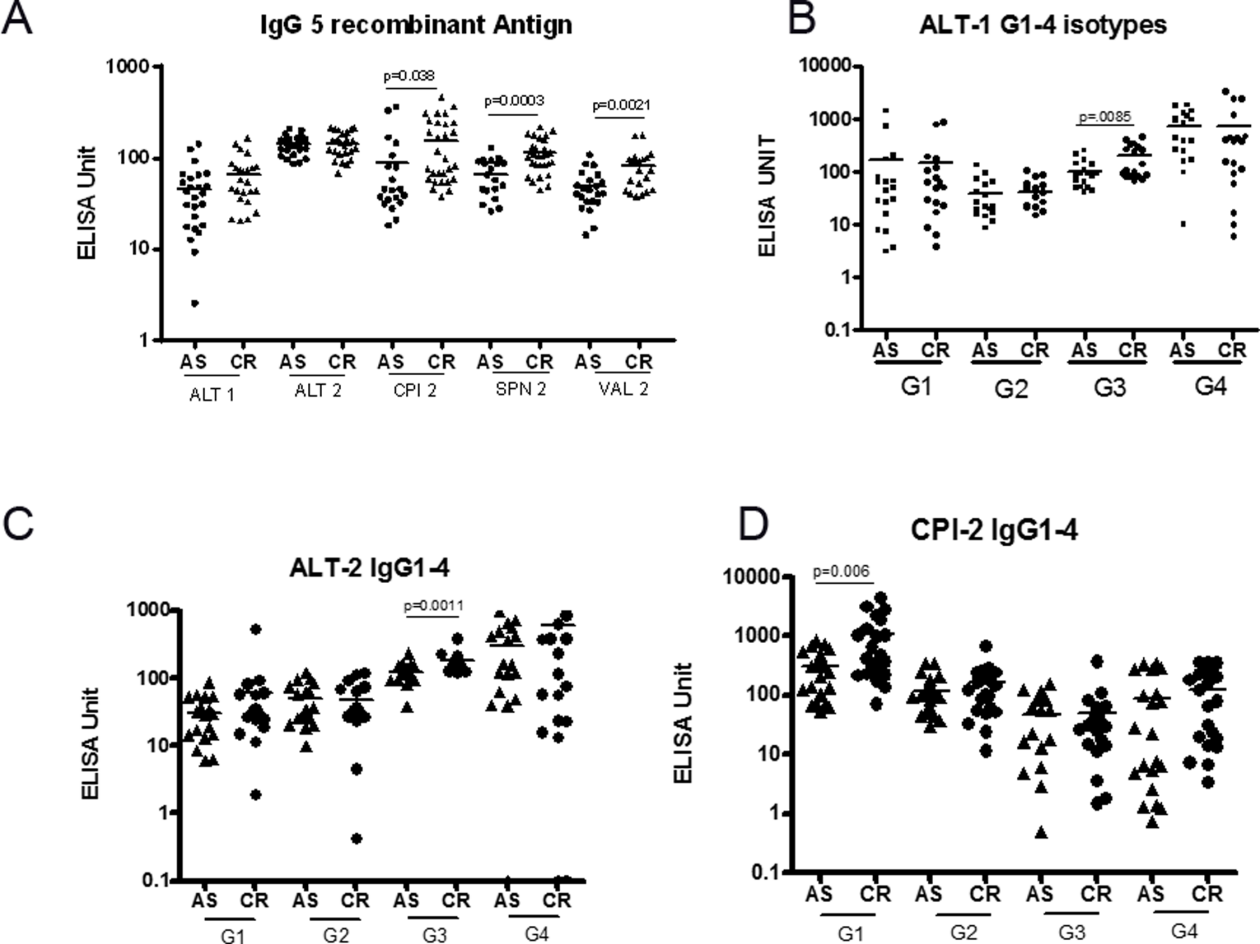
Profile of IgG and IgG subclasses against different filarial recombinant antigens. Plasma levels of IgG and IgG subclasses against stage specific recombinant filarial antigens were analyzed by ELISA. Whole IgG against five recombinant antigens such as ALT-1, ALT-2, CPI 2 SPN 2 and Val-2 (A) and IgG isotypes against ALT-1 (B), ALT-2 (C) and CPI-2 (D) were depicted. Mean of 30 and 31 samples were shown.

In order to identify the stage-specific antibody response, we measured the antibody levels against different recombinant filarial antigens expressed in particular stages of development. ALT-1 and ALT-2 are expressed by infective larval stage, SPN-2 is expressed by Mf, CPI-2 is expressed in the surface of adult filarial worms whereas Val-1 and Val-2 are expressed in all the developmental stages of filarial parasites [17]. Levels of IgG antibodies against SPN-2 and CPI-2 and Val-2 were significantly higher in CR group as compared to AS group (Fig 3A) supporting our previous finding that IgG responses against Mf and adult filarial antigen play a role in Mf clearance. Then we estimated different IgG isotype levels against the above mentioned recombinant filarial antigens. An increased IgG3 levels against larval stage ALT-1 and ALT-2 antigens and increased IgG1 response against adult stage CPI-2 was observed in CR cases whereas subclass response against SPN-2 or Val-1 remained comparable between AS and CR groups (Fig 3B–3D).

## Discussion

The factors responsible for deciding the fate of filarial pathogenesis remains unclear. Genetic traits, release of the bacterial endosymbiont *Wolbachia* and intensity of the host response have been implicated for the degree of filarial infection whereas precise mechanism responsible for parasite clearance and clinical manifestations between CR and AS group remains a matter of intense debate [18,19,20,21,22]. In this study, we demonstrated that the development of humoral response against filarial parasite and Mf is are associated with parasite clearance in CR cases.

A number of studies have been undertaken to understand the natural history of filarial infection. Meyrowitsch et. al undertook a 16 year follow up study of 542 subjects in Tanzania [22]. Similarly, Myung et. al conducted a 10 year follow up survey in southwest Benin [23]. Although the clinical manifestations remain unchanged in different groups of filarial patients, a decrease in Mf levels were observed in both the studies. Previously, we have demonstrated that a progressive decrease in prevalence and density of Mf in AS groups, whereas they continued to be CFA positive [13]. In this study, an increased humoral response against the developmental stages of filarial parasites (e.g. larvae, Mf) as well as adult worm, was observed in CR cases as compared to AS cases. Advanced understanding of filarial immunology stated that type-2 response plays the protective role against helminth parasites which is characterized by increased antibody response [24,25]. We also observed a correlation between increased antibody response and Mf clearance. So increased antibody levels in CR cases may be one of the important factors contributing to the removal of Mf from circulation.

Association between anti-sheath antibodies and antigenemia have been demonstrated [26]. In our previous study, we demonstrated that, anti-sheath antibodies are inversely correlated with CFA and based on the chronology of antibody response, we hypothesized that antibodies inhibit growth and development of larvae into mature worms [6]. Here, we demonstrated thatnot only anti-sheath antibodies rather antibody response to different developmental stages of the parasite have a positive association with Mf clearance. Although the mechanism of antigen clearance remains unclear, this study bolsters our hypothesis that broader anti-filarial immunity plays a protective role and is not restricted to anti-Mf immunity.

Different roles of IgG subclasses in filarial pathogenesis have been demonstrated. Anti-filarial IgG4 antibodies have been found to be associated with AS carriers whereas titers of IgG2 and IgG3 against filarial carbohydrates in CH cases have been observed [12,27,28]. The role of anti-filarial IgG subclass antibodies in CR cases remained unexplained. Our results demonstrated that CR cases exhibit increased titers of IgG1, IgG2, and IgG3 as compared to AS cases, whereas no difference in IgG4 subtype was observed. We and others demonstrated that increased IgG4 is a characteristic of AS cases [16,29], however, we found that high IgG4 titers are also present in CR cases in absence of circulating MF. Further research may clarify the association between microfilaremia and IgG4.

In conclusion, our study here deciphers a previously unknown role of anti-filarial antibodies in Mf clearance. The immune mechanisms responsible for parasite clearance in CR cases appears to be mediated by anti-filarial antibody generation. Further investigations may open up new avenues to understand the role of anti-Mf antibodies in parasite clearance.

## References

[1] Kilon AD (2014) Global programme to eliminate lymphatic filariasis: progress report, 2013. Wkly Epidemiol Rec 89: 409–418. 25243263

[2] WHO (2011) Global Programme to eliminate lymphatic filariasis: progress report on mass drug administration, 2010. Wkly Epidemiol Rec 86: 377–388. 21887884

[3] Taylor MJ, Hoerauf A, Bockarie M (2010) Lymphatic filariasis and onchocerciasis. Lancet 376: 1175–1185. doi: 10.1016/S0140-6736(10)60586-7 20739055 10.1016/S0140-6736(10)60586-7

[4] Sabesan S, Vanamail P, Raju K, Jambulingam P (2010). Lymphatic filariasis in India: Epidemiology and control measures. J Postgrad Med;56:232–2385. doi: 10.4103/0022-3859.68650 20739779 10.4103/0022-3859.68650

[5] Mishra R, Sahoo PK, Mishra S, Achary KG, Dwibedi B, et al (2014) Bancroftian filariasis: circulating B-1 cells decreased in Mf carriers and correlate with immunoglobulin M levels. Parasite Immunol 36: 207–217. doi: 10.1111/pim.12105 24495228 10.1111/pim.12105

[6] Ravindran B, Satapathy AK, Sahoo PK, Babu Geddam JJ (2000) Protective immunity in human Bancroftian filariasis: inverse relationship between antibodies to Mfl sheath and circulating filarial antigens. Parasite Immunol 22: 633–637. 11123755 10.1046/j.1365-3024.2000.00347.x

[7] Jawaharlal JP, Madhumathi J, Prince RP, Kaliraj P (2014) Repeat region of Brugia malayi sheath protein (Shp-1) carries Dominant B epitopes recognized in filarial endemic population. Acta Parasitol 59: 454–458. doi: 10.2478/s11686-014-0270-y 25119360 10.2478/s11686-014-0270-y

[8] Paciorkowski N, Shultz LD, Rajan TV (2003) Primed peritoneal B lymphocytes are sufficient to transfer protection against Brugia pahangi infection in mice. Infect Immun 71: 1370–1378. doi: 10.1128/IAI.71.3.1370-1378.2003 12595454 10.1128/IAI.71.3.1370-1378.2003 PMC148870

[9] Shenoy RK, Rakesh PG, Baldwin CI, Denham DA (1996) The sheath of the Mf of Brugia malayi from human infections has IgG on its surface. Parasitol Res 82: 382–384. 8740559 10.1007/s004360050132

[10] Zipperer GR, Arumugam S, Chirgwin SR, Coleman SU, Shakya KP, Klei TR (2013) Brugia pahangi: immunization with early L3 ES alters parasite migration, and reduces microfilaremia and lymphatic lesion formation in gerbils (Meriones unguiculatus). Exp Parasitol 135: 446–455. doi: 10.1016/j.exppara.2013.08.007 23981910 10.1016/j.exppara.2013.08.007 PMC3845383

[11] Sahoo PK, Geddam JJ, Satapathy AK, Mohanty MC, Ravindran B (2000) Bancroftian filariasis: prevalence of antigenaemia and endemic normals in Orissa, India. Trans R Soc Trop Med Hyg 94: 515–517. 11132379 10.1016/s0035-9203(00)90070-1

[12] Mohanty MC, Satapathy AK, Sahoo PK, Ravindran B (2001) Human bancroftian filariasis—a role for antibodies to parasite carbohydrates. Clin Exp Immunol 124: 54–61. doi: 10.1046/j.1365-2249.2001.01484.x 11359442 10.1046/j.1365-2249.2001.01484.x PMC1906036

[13] Sahoo PK, Babu Geddam JJ, Satapathy AK, Mohanty MC, Das BK, Acharya AS, et al (2002) Bancroftian filariasis: a 13-year follow-up study of asymptomatic Mfe carriers and endemic normals in Orissa, India. Parasitology 124: 191–201. 11862995 10.1017/s0031182001001007

[14] Sauerbrei A, Farber I, Brandstadt A, Schacke M, Wutzler P (2004) Immunofluorescence test for sensitive detection of varicella-zoster virus-specific IgG: an alternative to fluorescent antibody to membrane antigen test. J Virol Methods 119: 25–30. doi: 10.1016/j.jviromet.2004.02.012 15109817 10.1016/j.jviromet.2004.02.012

[15] Dafa'alla TH, Ghalib HW, Abdelmageed A, Williams JF (1992) The profile of IgG and IgG subclasses of onchocerciasis patients. Clin Exp Immunol 88: 258–263. 1572089 10.1111/j.1365-2249.1992.tb03070.x PMC1554306

[16] Jaoko WG, Simonsen PE, Meyrowitsch DW, Estambale BB, Malecela-Lazaro MN, Michael E. (2006) Filarial-specific antibody response in East African bancroftian filariasis: effects of host infection, clinical disease, and filarial endemicity. Am J Trop Med Hyg 75: 97–107. 16837715

[17] Hewitson JP, Harcus Y, Murray J, van Agtmaal M, Filbey KJ, Grainger JR, et al (2001) Proteomic analysis of secretory products from the model gastrointestinal nematode Heligmosomoides polygyrus reveals dominance of venom allergen-like (VAL) proteins. J Proteomics 74: 1573–1594. 10.1016/j.jprot.2011.06.002 PMC4794625 21722761

[18] Arndts K, Deininger S, Specht S, Klarmann U, Mand S, Adjobimey T, et al (2012) Elevated adaptive immune responses are associated with latent infections of Wuchereria bancrofti. PLoS Negl Trop Dis 6: e1611 doi: 10.1371/journal.pntd.0001611 22509424 10.1371/journal.pntd.0001611 PMC3317915

[19] Pfarr KM, Debrah AY, Specht S, Hoerauf A (2009) Filariasis and lymphoedema. Parasite Immunol 31: 664–672. doi: 10.1111/j.1365-3024.2009.01133.x 19825106 10.1111/j.1365-3024.2009.01133.x PMC2784903

[20] Mishra R, Panda SK, Sahoo PK, Bal MS, Satapathy AK (2017) Increased Fas ligand expression of peripheral B-1 cells correlated with CD4+ T-cell apoptosis in filarial-infected patients. Parasite Immunol 39. 10.1111/pim.12421 28208221

[21] Cuenco KT, Ottesen EA, Williams SA, Nutman TB, Steel C (2009) Heritable factors play a major role in determining host responses to Wuchereria bancrofti infection in an isolated South Pacific island population. J Infect Dis 200: 1271–1278. doi: 10.1086/605844 19754310 10.1086/605844

[22] Meyrowitsch DW, Simonsen PE, Makunde WH (1995) A 16-year follow-up study on bancroftian filariasis in three communities of north-eastern Tanzania. Ann Trop Med Parasitol 89: 665–675. 8745941 10.1080/00034983.1995.11813000

[23] Myung K, Massougbodji A, Ekoue S, Atchade P, Kiki-Fagla V, Klion AD (1998) Lymphatic filariasis in a hyperendemic region: a ten-year, follow-up panel survey. Am J Trop Med Hyg 59: 222–226. 9715936 10.4269/ajtmh.1998.59.222

[24] Panda SK, Kumar S, Tupperwar NC, Vaidya T, George A, Rath S et al (2012) Chitohexaose activates macrophages by alternate pathway through TLR4 and blocks endotoxemia. PLoS Pathog 8: e1002717 doi: 10.1371/journal.ppat.1002717 22654663 10.1371/journal.ppat.1002717 PMC3359989

[25] Turner JD, Langley RS, Johnston KL, Gentil K, Ford L, Wu B et al (2009) Wolbachia lipoprotein stimulates innate and adaptive immunity through Toll-like receptors 2 and 6 to induce disease manifestations of filariasis. J Biol Chem 284: 22364–22378. doi: 10.1074/jbc.M901528200 19458089 10.1074/jbc.M901528200 PMC2755959

[26] Simonsen PE, Meyrowitsch DW (1998) Bancroftian filariasis in Tanzania: specific antibody responses in relation to long-term observations on microfilaremia. Am J Trop Med Hyg 59: 667–672. 9840579 10.4269/ajtmh.1998.59.667

[27] Joseph SK, Verma SK, Sahoo MK, Sharma A, Srivastava M, Reddy MV et al (2012) IgG subclass responses to proinflammatory fraction of Brugia malayi in human filariasis. Indian J Med Res 135: 650–655. 22771594 PMC3401695

[28] Adjobimey T, Hoerauf A (2010) Induction of immunoglobulin G4 in human filariasis: an indicator of immunoregulation. Ann Trop Med Parasitol 104: 455–464. doi: 10.1179/136485910X12786389891407 20863434 10.1179/136485910X12786389891407 PMC3065634

[29] Ravindran B, Satapathy AK, Sahoo PK (1994) Bancroftian filariasis-differential reactivity of anti-sheath antibodies in Mfe carriers. Parasite Immunol 16: 321–323. 7970869 10.1111/j.1365-3024.1994.tb00355.x

